# Changes in Pathological Complete Response Rates after Neoadjuvant Chemotherapy for Breast Carcinoma over Five Years

**DOI:** 10.1155/2016/4324863

**Published:** 2016-06-13

**Authors:** Daniel C. McFarland, Jessica Naikan, Mariya Rozenblit, John Mandeli, Ira Bleiweiss, Amy Tiersten

**Affiliations:** ^1^Department of Medicine, Memorial Sloan Kettering Cancer Center, West Harrison, NY 10406, USA; ^2^Department of Pathology, Icahn School of Medicine at Mount Sinai, New York, NY 10029, USA; ^3^New York University, New York, NY 10016, USA; ^4^Department of Preventive Medicine, Icahn School of Medicine at Mount Sinai Hospital, New York, NY 10029, USA; ^5^Division of Hematology/Oncology, Tisch Cancer Institute, Icahn School of Medicine at Mount Sinai, New York, NY 10029, USA

## Abstract

Historically, neoadjuvant chemotherapy (NACT) was extrapolated from adjuvant regimens. Dual HER2 blockade and the introduction of carboplatin for triple negative breast cancers (TNBC) emerged by December 2013 and have improved pathological complete response (pCR) rates. The objective of this study was to assess the pCR rates before and after the introduction of these new neoadjuvant regimens.* Materials and Methods.* Stage I–III breast cancer patients who received NACT were analyzed for rates of pCR by clinical characteristics (i.e., age, BMI, axillary lymphadenopathy, and histologic subtype), by time period (1 = 3/2010–11/2013, 2 = 12/2013–3/2015), and by type of chemotherapy (e.g., anthracycline/taxane only, carboplatin-containing, and HER2 blockade).* Results.* 113 patients received NACT. Overall pCR rate was 26.5 percent (*n* = 30). The pCR rate increased from 14% to 43.1% (*p* = 0.001) from time period 1 to time period 2 and were associated with HER2 positivity (*p* = 0.003), receiving treatment during time period 2 (*p* = 0.001) and using an anthracycline/taxane plus additional agent type of regimen (*p* = 0.004).* Conclusions.* Our study revealed a significant difference in rates of pCR over five years. Window of opportunity trials and other trials that utilize pCR analysis should be encouraged.

## 1. Introduction

Neoadjuvant chemotherapy (NACT) was initially developed as a component of combined modality treatment for locally advanced breast cancer (LABC) that either was inoperable at presentation or required extended radical surgery [1]. The landmark trial, National Surgical Adjuvant Breast Project (NSABP) B-18, found no differences in disease-free survival (DFS) or overall survival (OS) based on the timing of chemotherapy relative to surgery in operable breast cancer patients but found that pCR correlated with DFS and OS. Following this trial, NAC was also used to increase the rate of breast conserving surgery.

Pathologic complete response rate (pCR) after neoadjuvant chemotherapy differs considerably across breast cancer subtypes [2–5]. Obtaining a pCR after neoadjuvant chemotherapy appears to have the strongest association with survival for patients with either HER2 overexpressed tumors or triple negative breast cancer (TNBC). The Neoadjuvant Herceptin (NOAH) trial demonstrated an absolute improvement in pCR of 20% with the addition of trastuzumab that translated into a 36% risk reduction in death at 5 years [6]. A large meta-analysis of 12 international neoadjuvant clinical trials confirmed improved survival, particularly among patients with TNBC and human epidermal growth factor receptor 2 (HER2) positive subtypes [7].

Until recently, neoadjuvant treatments have essentially been extrapolated from adjuvant treatment regimens. Pertuzumab is a recombinant humanized monoclonal antibody that targets the extracellular domain of the HER2 protein and blocks ligand-dependent heterodimerization of HER2 with other HER family members leading to cell growth arrest and apoptosis [8]. It may have a synergistic effect with trastuzumab and provide for dual HER2 blockade [9]. The FDA approved the use of pertuzumab in the neoadjuvant setting to be used along with trastuzumab and chemotherapy on September 30th, 2013, thus affording the opportunity of dual HER2 blockade in the neoadjuvant setting for patients with HER2 overexpressing breast cancers. Pertuzumab was the first FDA approved drug specifically for neoadjuvant use based on a pCR endpoint in the phase II NEOSPHERE with additional data from the TRYPHENA [10].

In December 2013, results from CALGB 40603 (Alliance) and I SPY 2 were released at the San Antonio Breast Cancer Symposium showing higher pCR rates among TNBC patients treated with a carboplatin-containing regimen in the neoadjuvant setting [11, 12]. The rationale for the use of platinum in the TNBC neoadjuvant setting has been its particular sensitivity to chemotherapy in general and specifically platinum agents [13].

We investigated our single-institution experience of obtaining a pCR after neoadjuvant chemotherapy over the last five years. Specifically, we evaluated response differences among breast cancer subtypes. We hypothesized that pCR rates have increased since the presentation of neoadjuvant dual HER2 therapy and carboplatin to treat TNBC.

## 2. Methods

This was a retrospective single-center analysis of stage I–III breast cancer patients who were treated with neoadjuvant chemotherapy from March 2010 until March 2015. The Institutional Review Board at the Icahn School of Medicine at the Mount Sinai Hospital approved this study. Patients were identified by the Pathology Department database at the Icahn School of Medicine at Mount Sinai Hospital. Patients were included only if their pre- and postneoadjuvant tissue specimens were available and their neoadjuvant regimens could be obtained in our hospital records. Patient characteristics were obtained via retrospective hospital-based chart review and included age at diagnosis, date of diagnosis, gender, BMI, tumor size on imaging, clinical axillary lymphadenopathy (i.e., biopsy proven), histologic grade, immunohistochemistry (IHC) for estrogen/progesterone expression, HER2 expression via IHC or fluorescent in situ hybridization (FISH), neoadjuvant chemotherapeutic regimen, therapy completion (yes/no), and pathological determination of pCR status. pCR was defined as having no residual invasive carcinoma in the breast and no tumor in the axillary lymph nodes. Isolated tumor cells (ITC) were allowed in the determination of pCR. Breast cancer pathologists performed all pathologic evaluations. IHC analyses were performed on formalin-fixed paraffin-embedded tissue sections Positive ER and PR status was defined as at least 5% of tumor cells with nuclear staining. One patient had ER expression of 1% and was treated as a TNBC and obtained a pCR. Tumors were considered HER2 positive with a score of 3+ on IHC and/or a FISH ratio of greater than 2.0 [14].

All neoadjuvant treatment regimens included anthracycline and/or taxane. For the purposes of this review, treatments were categorized based on the use of regimens containing anthracycline and/or taxane only (group 1), those regimens that included carboplatin (group 2) and those that contained dual HER2 blockade (group 3). Carboplatin-based regimens were only used in the treatment of TNBC and therefore did not overlap with the use of dual HER2 blockade. All patients completed all NACT cycles without any significant delays.

A time period variable was created based on the date of diagnosis and subsequent treatment either before or after December 1st, 2013 (time period 1 versus time period 2). All identified cases in time period 1 began their treatments prior to December 2013 and all cases in time period 2 began treatment after December 2013. This date of distinction was chosen based on emerging data at that time for the neoadjuvant use of carboplatin for the treatment of TNBC and the use of dual HER2 blockade, specifically with trastuzumab and pertuzumab, for the treatment of HER2 containing breast cancers.

### 2.1. Statistical Analysis

The primary objective of this retrospective study was to assess the rate of pCR after the use of neoadjuvant chemotherapy and to explore associations between tumor characteristics (size, receptor expression, and histology), patient characteristics (age, BMI, and clinical LAD), and treatment characteristics (standard versus carboplatin versus dual HER2 containing regimens). pCR is defined above. Continuous variables were summarized using mean and standard deviations, and categorical variables were summarized using frequency and percentages. Chi squared tests were used to assess the bivariate associations between categorical variables. Independent *t*-tests were used to assess the bivariate associations between continuous variables and the outcome dependent variable, pCR. A binomial logistic regression model was created for the dichotomous outcome pCR (yes/no) that incorporated statistically significant independent variables in the bivariate analysis to 0.1. Statistical procedures were performed using the SPSS version 22 software (SPSS, Chicago, IL, 2013) and statistical tests were two-tailed with 5% significance level.

## 3. Results

One hundred and thirteen patients who received NACT were reviewed in total. The cohort is described in Table 1 and was categorized as hormone receptor positive only (*n* = 33), HER2 positive (*n* = 43), and TNBC (*n* = 37) with an overall pCR of 26.5 percent (*n* = 30) for the entire cohort. By breast cancer subtype, pCR rates were as follows: hormone receptor positive only 12.1%, HER2 positive 41.9%, and TNBC 21.6%. The average age of the cohort was 51.14 (SD 13.1), body mass index (BMI) 27.83 (SD 7.4), and tumor size 3.35 cm (SD 2.2). Sixty-five (57.5%) patients had axillary lymphadenopathy found on exam or ultrasound and confirmed on biopsy. Sixty patients were included in time period 1 and 53 patients were included in time period 2. Eighty-five patients (75.2%) received an anthracycline and/or taxane only NACT and twenty-eight patients (24.8%) received other regimens, which were all delivered during time period 2. During time period 2, eight of 16 patients with TNBC received a carboplatin-containing regimen (50.0%) and 20 of 26 HER2+ breast cancer patients received a dual HER2 agent regimen (76.9%).

Several group differences were noted between time periods 1 and 2 (Table 1). The pCR rate after NACT increased from 14% (8 out of 60 cases) to 43.1% (22 out of 53 cases) (*p* = 0.001). Patient age dropped from 53.19 to 48.85 years (*p* = 0.09) and tumor size dropped from 3.43 to 2.49 cm on average (*p* = 0.01). HER2 positive cases increased from 17 to 26 (*p* = 0.04) and hormone receptor positive only cases dropped from 22 to 11 (*p* = 0.04) and TNBC cases dropped from 21 to 16 (*p* = 0.6). BMI and clinical lymphadenopathy did not vary significantly.

Table 2 highlights group differences between those patients who achieved a pCR after neoadjuvant chemotherapy and those who did not achieve a pCR. Overall, higher pCR rates were associated with HER2+ containing breast cancers (*p* = 0.003), being treated during time period 2 (*p* = 0.001) and using a nonanthracycline/taxane only regimen (e.g., carboplatin-containing or dual HER2 blockade) (*p* = 0.004). Hormone receptor positive only tumors were significantly associated with not achieving a pCR (*p* = 0.003). Anthracycline/taxane only regimens were used in 81% of cases that did not achieve a pCR and 50% of cases that did achieve a pCR. That is, the use of nonanthracycline/taxane only regimens was associated with achieving a pCR. Although only 28 out of 113 cases utilized a nonstandard chemotherapy, there was a higher likelihood of obtaining a pCR. The carboplatin-containing chemotherapy obtained a pCR in five out of eight cases (62.5%) and dual HER2 blockade achieved a pCR in 10 out of 20 cases (50.0%). Age, initial tumor size, BMI, and initial clinical lymphadenopathy were not significantly associated with pCR (Table 2).

The role of NACT and other predictive variables for obtaining a CR is borne out more clearly by multivariate binomial logistic regression (Table 3). Although univariate analysis found that time period, regimen, HER2 positivity, and hormone positive predicted pCR, multinomial logistic regression found that time period was the only variable significantly associated with the probability of achieving pCR. That is, NACT and the other predictive variables for obtaining a pCR (i.e., tumor type [HER2, hormone positive only]) no longer significantly predicted the probability of achieving a pCR in the multinomial logistic regression model presented in Table 3. Time period 2 was the only variable significantly associated with pCR (*p* = 0.03).

## 4. Discussion

Our study revealed a significant difference in rates of pCR achieved at a single institution over five years. Overall, the only variable that was significantly associated with pCR was when the patient was treated (i.e., after December 1st, 2013) and not clinical characteristics (i.e., tumor size, age, BMI, clinical LAD, and breast cancer subtype). However, there were significant clinical differences between time periods 1 and 2. A greater number of HER2 positive patients, fewer hormone receptor positive patients, and smaller tumors were selected for neoadjuvant therapy during time period 2. But, these characteristics did not appear to play a role in obtaining a pCR overall.

Our baseline rates of pCR during time period 1 (i.e., 8 out of 60 cases or 13.3% of NACT cases) approximated previously obtained pCR rates using standard NACT regimens [15]. Although the breast cancer subgroup numbers were relatively small, time period 2 revealed much higher rates of pCR for HER2+ and TNBC subgroups (HER2+ 50%, TNBC 62.5%). Our pCR rates after the introduction of dual HER2 blockade and carboplatin for TNBC are similar to previously reported pCR rates. For example, the addition of dual HER2 blockade appears to improve pCR rates by 16–19% regardless of combined chemotherapy type and rates of pCR based on a meta-analysis of six included trials that used dual HER2 inhibition [16]. Also, the addition of carboplatin to standard dose dense NACT was shown to improve pCR rates by 13–21% [12, 16, 17]. Our study only included pCR samples that had no residual disease in the axilla or lymph nodes; the pCR definition most strongly correlated with improvement in survival outcomes [10]. For aggressive breast cancer subtypes that achieve a pCR, the risk of death has been shown to decrease by 84% in TNBC, 92% in HER2+, and 71% in grade 3 hormone receptor positive/HER2-negative breast cancers in the CTNeoBC pooled analysis [7]. Our analysis will include a future analysis to determine relapse-free and overall survival.

There are several limitations to our retrospective analysis. Time period 2 contributory factors towards obtaining a pCR may also include a patient selection bias (i.e., smaller tumors, more HER2+ tumors and less hormone receptor only tumors, and perhaps younger age) that could have influence the rate at which pCR status was achieved. Also, although the sample size was sufficient to describe our endpoint of pCR over time, there were relatively small patient numbers to meaningfully analyze the associations of clinical and pathological characteristics with pCR. For these reasons, the actual association between the regimen and pCR versus the time period and pCR cannot be reliably determined.

This study shows improved rates of pCR after NACT at our single institution that was most strongly associated with when they were treated (i.e., time period 2). Improved rates of pCR coincided with several institutional changes in selecting neoadjuvant patients and the use of new regimens for HER2 positive breast cancer and TNBC. Although the numbers of subgroups were small, our analysis shows a large difference in rates of pCR based on breast cancer subtypes. Overall, the meaning of pCR is still not entirely well defined but appears to be a meaningful endpoint, especially for aggressive HER2 positive breast cancer and TNBC. Window of opportunity trials and other trials that utilize pCR analysis should be encouraged.

## Figures and Tables

**Table 1:** Cohort demographics and clinical characteristics.

Characteristic	Entire cohort *n* = 113	Time period 1: *n* = 60	Time period 2: *N* = 53	Test statistic
	Mean (SD)	Mean (SD)	Mean (SD)	*t* (*p*)
Age	51.1 (13.1)	53.19 (11.30)	48.85 (14.70)	1.72 (0.09)
Initial tumor size (cm)	3.35 (2.2)	3.43 (2.2)	2.49 (1.48)	2.51 (0.01)^*∗*^
BMI	27.8 (7.4)	28.81 (8.13)	26.7 (6.43)	1.50 (0.14)

	*N* (%)	*N* (%)	*N* (%)	*χ* ^2^ (*p*)

Clinical LAD	65 (57.5)	37 (61.7)	28 (52.8)	0.88 (0.35)
Hormonal receptor positive only	33 (29.2)	22 (36.7)	11 (20.7)	4.18 (0.04)^*∗*^
HER2+	43 (38.1)	17 (28.3)	26 (49.1)	4.92 (0.03)^*∗*^
TNBC	37 (32.7)	21 (35.0)	16 (30.2)	0.28 (0.60)
pCR overall	30 (26.5)	8 (13.3)	22 (41.5)	11.36 (0.001)^*∗∗*^

BMI: body mass index; HER2: human epidermal growth factor receptor 2; LAD: lymphadenopathy; NACT: neoadjuvant chemotherapy; pCR: pathological complete response; time period 1: before 12/2013; time period 2: after 12/2013; TNBC: triple negative breast cancer.

*Notes.* ^*∗∗*^ *p* < 0.01 and  ^*∗*^ *p* < 0.05.

**Table 2:** Clinical and pathological associations with pCR by univariate analysis. This table lists tumor characteristics (size, receptor expression, and histology), patient characteristics (age, BMI, and clinical LAD), and treatment characteristics (standard NACT versus carboplatin-containing NACT and dual HER2 blockade regimens) and their associations with pCR rates.

Characteristic	pCR = no	pCR = yes	Test statistic
	*n* = 83	*n* = 30	
	Mean (SD)	Mean (SD)	*t* (*p*)
Age	53.00 (12.8)	46.67 (13.2)	2.28 (0.51)
Initial tumor size (cm)	3.01 (2.0)	2.80 (1.8)	0.55 (0.59)
BMI	28.0 (7.9)	27.4 (6.1)	0.41 (0.37)

	*N* (%)	*N* (%)	*χ* ^2^ (*p*)

Clinical LAD			
(i) Yes	49 (63.6)	16 (57.1)	0.370 (0.545)
(ii) No	24 (36.4)	14 (42.9)	
Hormone rec only			
(i) Yes	29 (37.7)	4 (13.8)	6.00 (0.018)
(ii) No	54 (62.3)	26 (86.2)	
HER2+			
(i) Yes	25 (30.1)	18 (60.0)	8.57 (0.003)^*∗∗*^
(ii) No	58 (69.9)	12 (40.0)	
TNBC			
(i) Yes	29 (34.9)	8 (26.7)	0.34 (0.563)
(ii) No	54 (65.1)	22 (73.3)	
Time period			
(i) Before	49 (62.8)	8 (26.7)	11.36 (0.001)^*∗∗*^
(ii) After	29 (37.2)	22 (73.3)	
Regimen			
(i) Standard	58 (81.7)	15 (50)	10.99 (0.004)^*∗∗*^
(ii) Carboplatin-containing	3 (4.2)	5 (16.7)	
(iii) Dual HER2 blockade	10 (14.1)	10 (33.3)	

BMI: body mass index; HER2: human epidermal growth factor receptor 2; hormone rec only: hormone receptor only; LAD: lymphadenopathy; NACT: neoadjuvant chemotherapy; pCR: pathological complete response; TNBC: triple negative breast cancer.

*Notes*.  ^*∗∗*^ *p* < 0.01; row data does not match column data in all cases.

**Table 3:** Multivariate logistic regression model examining predictors of pathological complete response among female breast cancer patients treated with neoadjuvant chemotherapy (*N* = 111).

Variable	*B* (SE)	OR	Sig	95% CI	
				Lower	Upper
Hormone receptor positive only	−0.531 (0.74)	0.486	0.588	0.139	2.492
HER2+	0.809 (0.66)	2.247	0.223	0.611	8.259
Time period (after 12/2013)	1.379 (0.63)	3.97	0.03^*∗*^	1.146	13.753
Regimen (standard)			0.639		
Regimen (with carboplatin)	0.008 (0.76)	1.008	0.992	0.226	4.505
Regimen (with dual HER2 blockade)	0.818 (0.97)	2.266	0.401	0.336	15.290
Constant	−1.968 (0.84)	0.140	0.06		

*Notes*.  ^*∗*^ *p* < 0.05.
